# Custom total knee arthroplasty facilitates restoration of constitutional coronal alignment

**DOI:** 10.1007/s00167-020-06153-8

**Published:** 2020-07-17

**Authors:** Michel P. Bonnin, Lucas Beckers, Augustin Leon, Jules Chauveau, Jacobus H. Müller, Carsten O. Tibesku, Tarik Aït-Si-Selmi

**Affiliations:** 1 Centre Orthopédique Santy, Hôpital Privé Jean Mermoz, Ramsay Santé, Lyon, France; 2 ReSurg SA, Rue Saint Jean 22, 1260 Nyon, Switzerland; 3 KniePraxis, Straubing, Germany

**Keywords:** Custom TKA, Total knee replacement, Total knee arthroplasty, HKA, FMA, TMA, Coronal alignment, Patient-specific

## Abstract

**Purpose:**

To describe a strategy for coronal alignment using a computed tomography (CT) based custom total knee arthroplasty (TKA) system, and to evaluate the agreement between the planned and postoperative Hip–Knee–Ankle (HKA) angle, Femoral Mechanical Angle (FMA) and Tibial Mechanical Angle (TMA).

**Methods:**

From a consecutive series of 918 primary TKAs, 266 (29%) knees received CT-based posterior-stabilized cemented custom TKA. In addition to a preoperative CT-scan, pre- and post-operative radiographs of weight-bearing long leg, anterior–posterior and lateral views of the knee were obtained, on which the FMA, TMA and HKA angles were measured. CT-based three-dimensional (3D) models enabled to correct for cases with bony wear by referring to the non-worn areas and to estimate the native pre-arthritic angles. The alignment technique aimed to preserve or restore constitutional alignment (CA) within predetermined limits, by defining a ‘target zone’ based on three criteria: 1) a ± 3° (range 87°–93°) primary tolerance for the femoral and tibial resections; 2) a ± 2° secondary tolerance for component obliquity, extending the bounds for FMA and TMA (range 85°–95°); 3) a planned HKA angle range of 175°–183°. Agreement between preoperative, planned and postoperative measurements of FMA, TMA and HKA angle were calculated using intra-class correlation coefficients (ICC).

**Results:**

Preoperative radiograph and CT-scan measurements revealed that, respectively, 73 (28%) and 103 (40%) knees were in the ‘target zone’, whereas postoperative radiographs revealed that 217 (84%) TKAs were in the ‘target zone’. Deviation from the planned angles were − 0.5° ± 1.8° for FMA, − 0.5° ± 1.8° for TMA, and − 1.1° ± 2.1° for HKA angle. Finally, the agreement between the planned and achieved targets, indicated by ICC, were good for FMA (0.701), fair for TMA (0.462) and fair for HKA angle (0.472).

**Conclusion:**

Using this strategy for coronal alignment, 84% of custom TKAs were within the ‘target zone’ for FMA, TMA and HKA angles. These findings support the concepts of emerging personalized medicine technologies, and emphasise the importance of accurate strategies for preoperative planning, which are key to achieving satisfactory ‘personalised alignment’ that can further be improved by customisation of implant components.

**Level of evidence:**

IV.

**Electronic supplementary material:**

The online version of this article (10.1007/s00167-020-06153-8) contains supplementary material, which is available to authorized users.

## Introduction

Various knee alignment strategies for total knee arthroplasty (TKA) have been described and investigated, with the goals of improving functional outcomes and implant survival, but there remains little or no consensus about an optimal strategy. The different alignment philosophies can be considered as three main categories [39]: Systematic alignment (mechanical [21] or anatomical [20]), patient-specific alignment (kinematic [18]), and hybrid alignment (adjusted mechanical [41] or restricted kinematic [2]).

Mechanical alignment aims to restore a neutral tibiofemoral alignment, by making resections orthogonal to the tibial and femoral mechanical axes, regardless of a patient’s pre-arthritic constitutional alignment (CA) [4]. Recently, it has been suggested that maintaining a residual varus alignment does not compromise TKA survival [1, 34] and might improve functional outcomes [11, 27], whereas changing a preoperative varus to a postoperative valgus, or vice versa, could jeopardize functional outcomes [27]. By contrast, kinematic alignment aims to preserve or restore CA [4, 26, 29], by aligning implants to the native condylar and tibial joint lines, and by matching component thickness of the femoral condyles and tibia to that of resected bone [17, 19].

Although some studies have highlighted the limits of systematic neutral alignment [1, 11, 24], the concept of patient-specific alignment continues to raise some controversial issues: First, with conventional radiographic preoperative planning, it is difficult to differentiate between constitutional and arthritic deformities [4, 6, 22, 28]. Second, the thresholds for acceptable postoperative residual varus or valgus remain unknown [2, 24, 31, 35]. Third, component alignment and design are interrelated, and an individualized alignment associated with conventional implants can induce bone-implant mismatch or alter patellofemoral kinematics [31, 33, 37].

The theoretical benefits of custom TKA based on computed tomography (CT) reconstructions include the differentiation of constitutional versus arthritic bony deformities, as well as identification of the native femoral and tibial axes [11]. This concept has the potential to reduce bone-implant mismatch and preserve or restore CA within predetermined limits, to maintain the native overall phenotype while allowing correction of severe deformities [14–16]. The purpose of this study was to describe the strategy for coronal alignment using a CT-based custom TKA system and to evaluate the agreement between the planned and postoperative Hip–Knee–Ankle (HKA) angle, Femoral Mechanical Angle (FMA) and Tibial Mechanical Angle (TMA). The hypothesis was that custom TKA would enable achieving the intended coronal alignment in at least 80% of cases, irrespective of their preoperative deformity. The findings of this study would add to the evidence on emerging personalized medicine technologies, that can further be improved by customisation of implant components.

## Materials and methods

### Patients

**Fig. 1:**
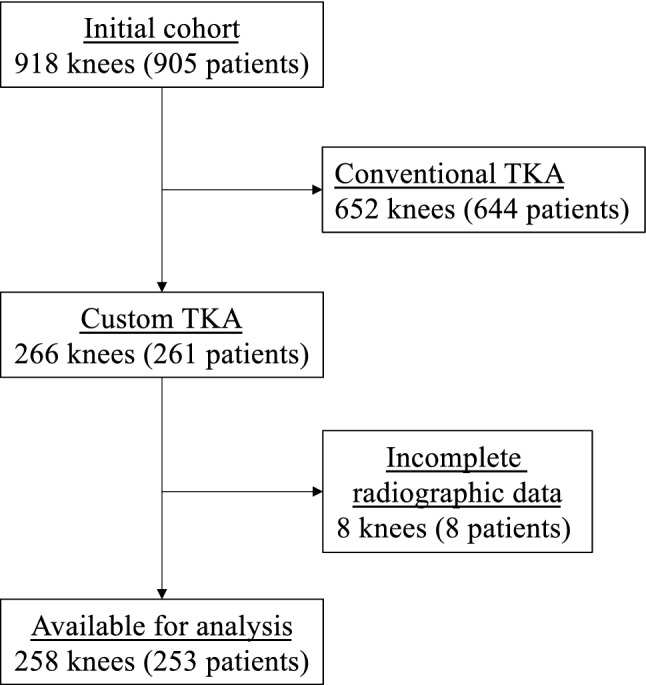
Flowchart of the study cohort

From a consecutive series of 918 knees (905 patients) that received primary TKA between January and December 2018 by three surgeons (MPB,TASS,COT) at two centres, 266 (29%) knees (261 patients) received CT-based postero-stabilized cemented custom TKA (Origin^®^ TKA, Symbios, Yverdon les bains, Switzerland). All patients had provided written informed consent for the use of their data and images for research and publishing purposes and the institutional review board approved the study in advance (IRB reference number: COS-RGDS-2019-10-004-BONNIN-M; Ramsay Santé Comité d’Ethique; + 33 (0)1 87 86 22 97; Dr Sylviane Olschwang). Exclusion criteria for a custom TKA were: severe coronal deformities > 15°, stiff knees with extension deficit > 15°, flexion range < 90°, severe medial laxity > 10°, or severe lateral laxity > 15°. The final decision for using a custom TKA was based on the logistics of an 8 week waiting-period required for the design and manufacturing processes. The indications for surgery were medial osteoarthritis (OA) in 213, lateral OA in 34, global OA in four, patellofemoral OA in eight and rheumatoid arthritis (RA) in seven knees. Eight knees were excluded due to unavailable postoperative long-leg radiographs, leaving a final cohort of 258 TKAs (118 men (five bilateral) and 135 women), with a mean age at index surgery of 70 ± 9 years (range 46–93 years) and a mean BMI of 29.5 ± 13 kg/m^2^ (range 18–44). Thirty knees had previous surgery: ligament reconstruction (*n* = 25), fixation of a distal femoral or proximal tibial fracture (*n* = 5), high tibial osteotomy (HTO, *n* = 6), or tibial tubercle transfer (*n* = 3) (Fig. 1).

### The custom TKA prosthesis

All patients received a CT-scan (64-slice multidetector scanner, Siemens^®^ Sensation, Munich, Germany) according to the manufacturer’s protocol 8 weeks before surgery, on which the FMA, TMA and HKA angles were measured (Knee-Plan^®^ software, Symbios, Yverdon les bains, Switzerland) to an accuracy of 1° [15]. Three-dimensional (3D) models enabled to correct for cases with bony wear by referring to the non-worn areas and to estimate the native pre-arthritic angles [11]. The Origin® prosthesis is CE-marked and its design is based on a 3D analysis of bony anatomy, arthritic deformities and native limb alignment, as captured by the preoperative CT-scans. The femoral component reproduced the contours, sagittal radii of curvature and joint line obliquity of the native femur. The tibial baseplate reproduced the contours of the native tibial plateau, and the polyethylene insert had up to 2 mm difference in thickness between the medial and lateral compartments. Manufacturing of the femoral component was based on a conventional Cobalt–Chromium casting process, followed by machining and polishing, whereas the tibial baseplate was machined from Titanium alloy. Single-use patient-specific cutting guides were manufactured from polyamide using additive manufacturing technology.

### Alignment strategy

**Fig. 2:**
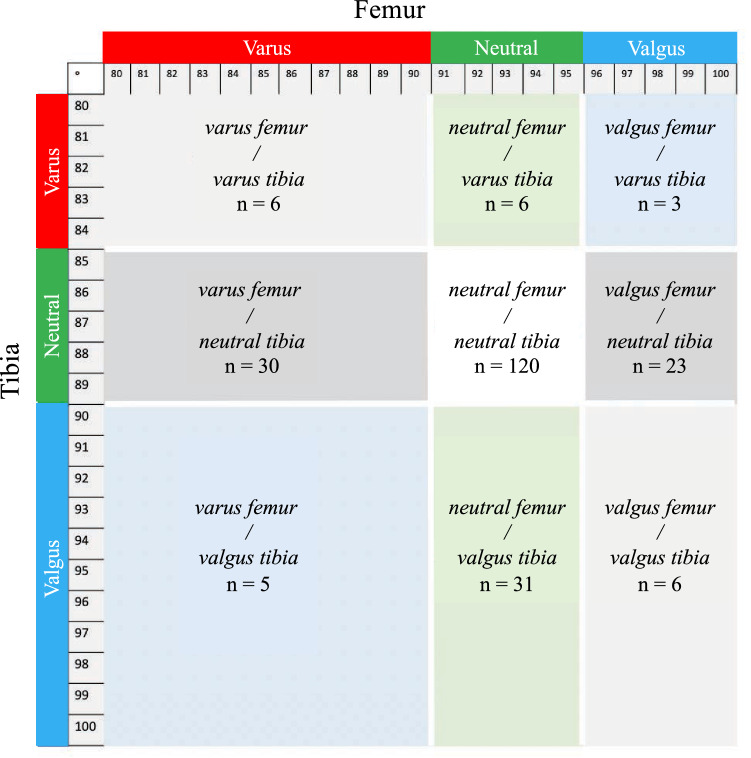
This matrix represents FMA on the horizontal axis and TMA on the vertical axis. Varus femurs were defined as FMA < 91° and Valgus femurs as FMA > 95°; Varus tibias were defined as TMA < 85° and Valgus tibias as TMA > 89°. Overall limb alignment was considered in varus if HKA angle < 177° and in valgus if HKA angle > 183°. Finally nine morphotypes were defined based on FMA and TMA

Based on the classification of Hirschmann et al. [15, 16], which distinguishes 25 categories of HKA coronal alignment (five femoral phenotypes $\times$ five tibial phenotypes), the present study considered nine categories of HKA coronal alignment (three femoral phenotypes $\times$ three tibial phenotypes) (Fig. 2). This simplification was mainly achieved by disregarding the extent of varus or valgus deformity (minor if < 4.5° or major if > 4.5°) within each bone, and by widening the range of what is considered to be neutral FMA (91°–95° instead of 91.5°–94.5°), and what is considered to be neutral TMA (85°–89° instead of 85.5°–88.5°).

**Fig. 3:**
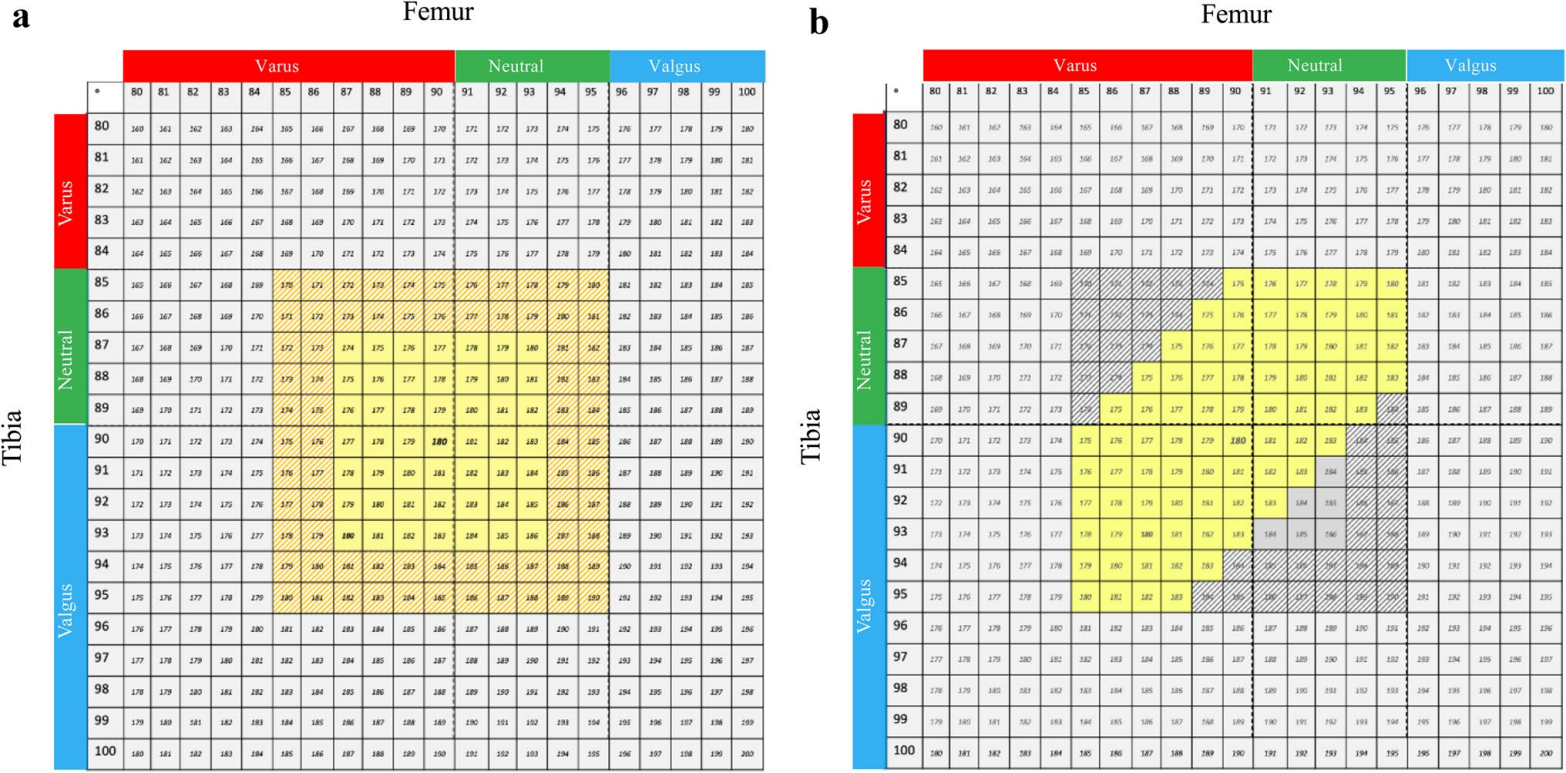
**a** Matrix shows the alignment possibilities offered by the Origin^®^ system. A primary tolerance of ± 3° for the femoral and tibial cuts, which were planned to achieve a range of mechanical angles from 87° to 93° (yellow area). A secondary tolerance of ± 2° for implant obliquity (polyethylene insert and femoral condyles), which extended the total range of mechanical angles from 85° to 95° (Orange area). **b** ‘Target zone’ (Yellow area) respecting the 85° to 95° range for FMA and TMA, and the 175° to 183° range for HKA angle

The alignment technique for the Origin^®^ TKA aims to preserve or restore CA within predetermined limits, allow correction of severe deformities, and maintain the native overall phenotype, by defining a ‘target zone’ based on three criteria (Fig. 3):

1. A primary tolerance of ± 3° for the femoral and tibial cuts, which were planned to achieve a range of mechanical angles from 87°–93°, depending on the pre-arthritic phenotype [12, 35, 36].
2. A secondary tolerance of ± 2° for implant obliquity (polyethylene insert and femoral condyles), which extended the total range of mechanical angles from 85°–95°, to remain as close as possible to the pre-arthritic phenotype [32].
3. A planned HKA angle within the range of 175°–183° [27].

**Fig. 4:**
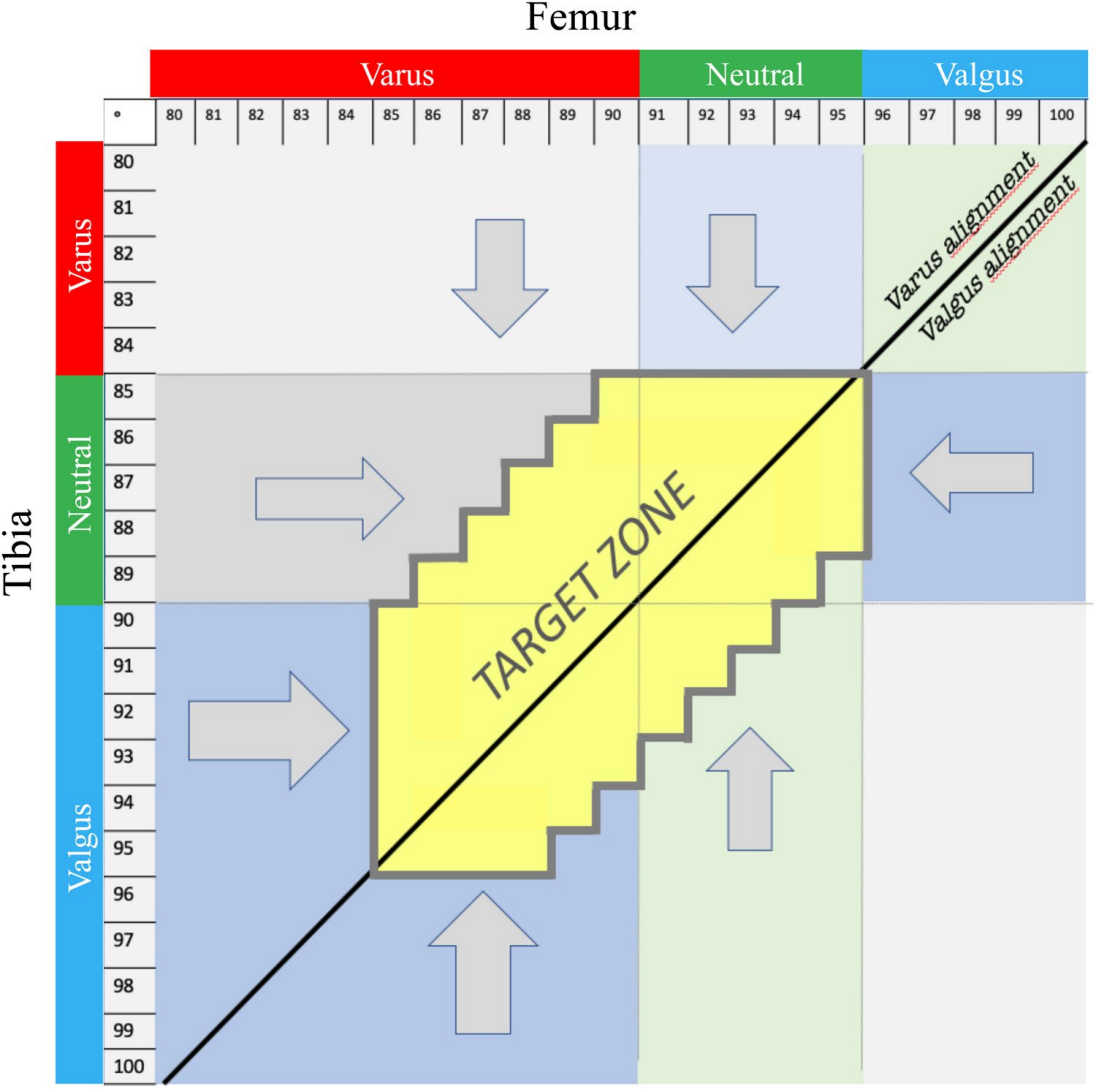
When the preoperative FMA and TMA are within the ‘target zone’, the constitutional alignment is maintained postoperatively. When the preoperative FMA and TMA are outside the ‘target zone’, the alignment is corrected

**Fig. 5:**
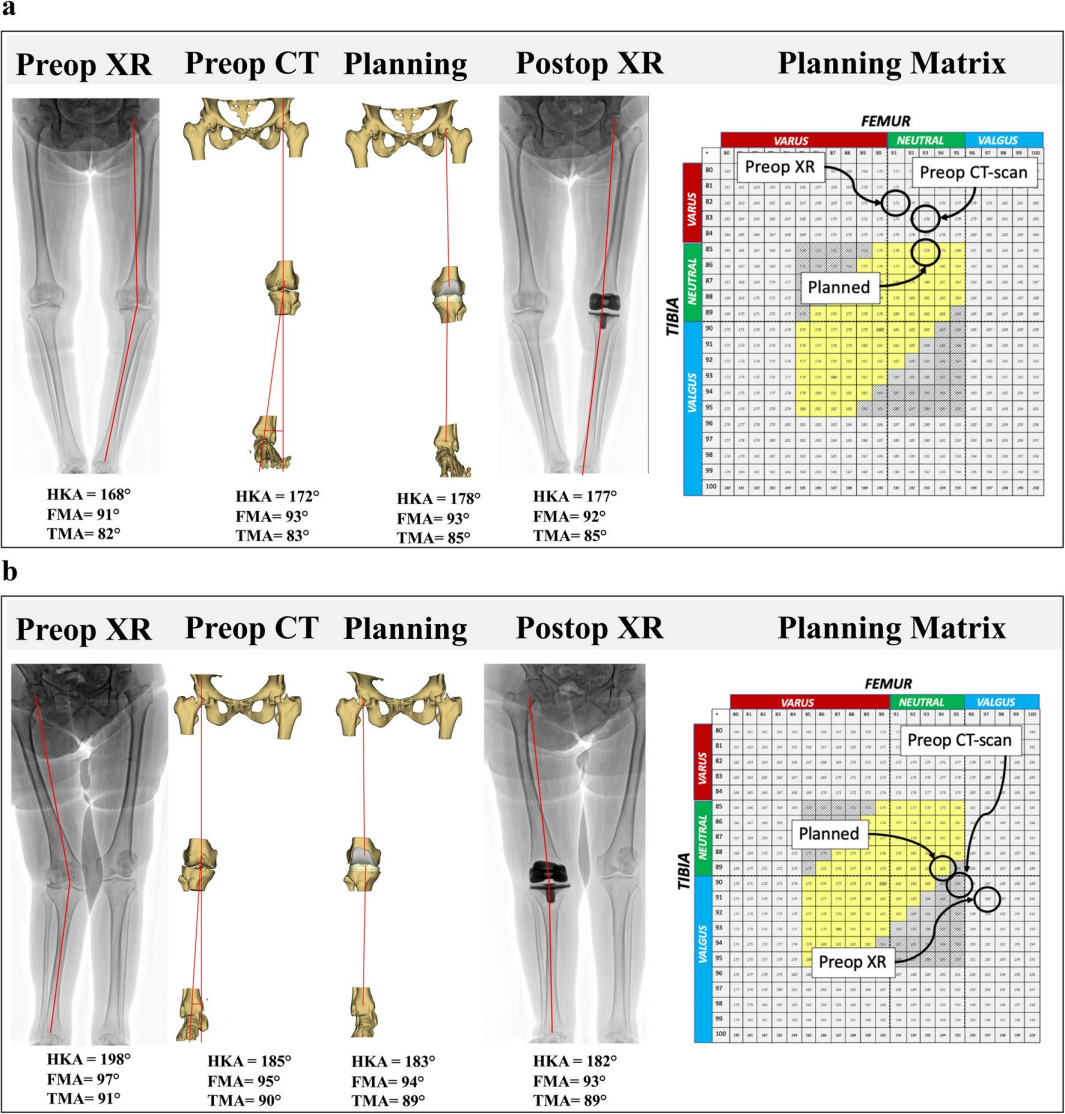
**a** In this patient, the global radiograph varus deformity is a combination of arthritic deformity (bone wear and laxity) and constitutional deformity (HKA angle = 168°). Constitutional alignment is outside the ‘target zone’ (FMA = 91° and TMA = 82°). The planning corrects the deformity to the ‘target zone’ (FMA = 92°, TMA = 85°, HKA angle = 177°). **b** In this patient, the global radiograph valgus deformity is a combination of arthritic deformity (bone wear and laxity) and constitutional deformity (HKA angle = 198°). Constitutional alignment is outside the ‘target zone’ (FMA = 97° and TMA = 91°). The planning corrects the deformity to the ‘target zone’ (FMA = 93°, TMA = 89°, HKA angle = 182°)

The application of the three criteria resulted in a ‘target zone’ (Fig. 4). When the preoperative FMA and TMA were inside the ‘target zone’, the planned alignment should correspond to CA, while when the preoperative FMA and TMA were outside of the ‘target zone’, the planned alignment was corrected to the closest configuration within the ‘target zone’, to maintain the native overall phenotype (Fig. 5).

### Surgical technique

All patients were operated using a medial parapatellar approach. Femoral and tibial resections were made using the custom cutting guides by a ‘femur-first’ technique. Soft tissue balance was then evaluated with a dynamic spacer, and if necessary, the level of tibial resection was adjusted by making a ‘recut’ using a dedicated guide. Once all bone surfaces were prepared, the tibia and then the femur and patella were cemented. Immediate full weight-bearing was authorised, and rehabilitation began on the same day of surgery.

### Radiographic analysis

Pre- and postoperative radiographs of weight-bearing long leg, anterior–posterior and lateral views of the knee and a skyline view of the patella were obtained for each patient. Radiographic follow-up occurred at 4 months. Pre- and postoperative alignment was measured on the weight-bearing long-leg radiographs: The mechanical Femoral Axis (mFA) was defined as the line joining the centre of the femoral head and the centre of the intercondylar notch. The FMA was measured medially between the mFA and the distal condylar line. The mechanical Tibial Axis (mTA) was defined as the line joining the middle of the tibial spines and the centre of the tibiotalar joint. The TMA was then measured medially between the tibial joint line and the mTA. Finally, the HKA angle was measured medially between mFA and mTA. Radiographic measurements were performed (MPB) on an online picture archiving and communication system (GXD5 PACS, groupe NGI, Lyon, France) with a measurement accuracy of 0.1°, and rounded to the nearest whole number.

### Statistical analysis

Shapiro–Wilk tests were used to assess the normality of data distributions. For non-Gaussian quantitative data, differences between groups were evaluated using Wilcoxon rank sum tests (Mann–Whitney *U* test) and Kruskal–Wallis tests. For categorical data, differences between groups were evaluated using Chi-squared tests. Agreement between preoperative, planned and postoperative measurements of FMA, TMA and HKA angle were calculated using intra-class correlation coefficients (ICC), which can be interpreted as follows: < 0.40 poor; 0.40–0.59 fair; 0.60–0.74 good; 0.75–1.00 excellent. A second observer (LB) repeated radiographic measurements of pre- and post-operative FMA, TMA and HKA angles for 25 (10%) knees. The interobserver agreement calculated using ICC was excellent (ICC 0.774–0.994) for all measurements. Statistical analyses were performed using R version 3.3.2 (R Foundation for Statistical Computing, Vienna, Austria). *p* values < 0.05 were considered statistically significant.

## Results

Preoperative coronal alignment as measured on CT-scans and radiographs were, respectively, 92.7° ± 2.5° and 92.2° ± 2.6° (n.s.) for FMA, 87.0° ± 2.7° and 86.5° ± 3.0° (*p* = 0.038) for TMA, and 176.9° ± 5.0° and 175.6° ± 6.2° (*p* < 0.001) for HKA angle (Table 1). The most common phenotype, as measured from the preoperative CT-scans, was ‘*neutral femur and neutral tibia*’ (*n* = 120, 47%), and the least common was ‘*valgus femur and varus tibia*’ (*n* = 3, 1%) (Fig. 2). The proportion of knees that were in the ‘target zone’ according to preoperative radiograph and CT-scan measurements were, respectively, 73 (28%) and 103 (40%).

**Table 1:** Preoperative coronal alignment according to radiograph and CT measurements

	Mean ± SD ICC	(Range) (95% CI)	*p* values (Radiograph vs. CT)
FMA			
Radiograph (deg)	92.2 ± 2.6	(84–99)	
CT (deg)	92.7 ± 2.5	(87–99)	n.s
Delta (deg)	0.4 ± 1.5	(− 4–5)	
Agreement	0.827	(0.767–0.870)	
TMA			
Radiograph (deg)	86.5 ± 3.0	(79–95)	
CT (deg)	87.0 ± 2.7	(80–94)	0.038
Delta (deg)	0.5 ± 1.6	(− 4–7)	
Agreement	0.830	(0.764–0.876)	
HKA angle			
Radiograph (deg)	175.6 ± 6.2	(162–195)	
CT (deg)	176.9 ± 5.0	(165–192)	< 0.001
Delta (deg)	1.3 ± 2.8	(− 11–13)	
Agreement	0.859	(0.762–0.910)	

*FMA* femoral mechanical angle; *TMA* tibial mechanical angle, *HKA* hip knee ankle; *CT* computed tomography; *SD* standard deviation; *ICC* intra-class correlation cofficient; *CI* confidence interval; *deg* degree; *n.s* not signifcant

**Fig. 6:**
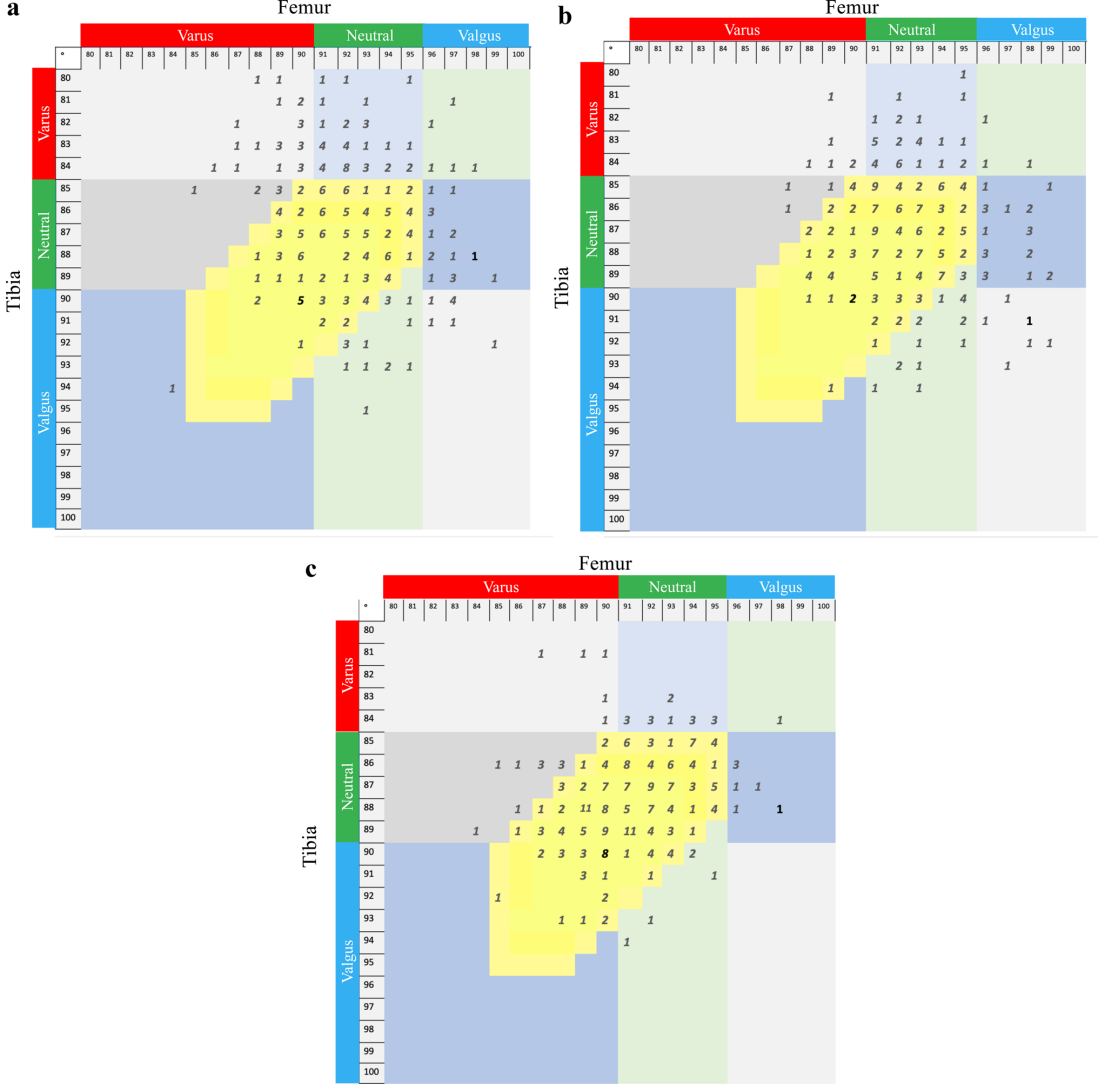
258 knees of the series are included in these matrices with FMA and TMA measured on: **a** preoperative long-leg radiographs, **b** preoperative CT-scans, and **c** postoperative long-leg radiographs

Deviation between the planned and postoperative angles were − 0.5° ± 1.8° for FMA, − 0.5° ± 1.8° for TMA, and − 1.1° ± 2.1° for HKA angle (Table 2). A total of 217 (84%) TKAs were in the ‘target zone’, based on the postoperative radiograph measurements (Fig. 6). Finally, the agreement between the planned and achieved targets, indicated by ICC, were good for FMA, but fair for TMA and HKA angle.

**Table 2:** Planned and postoperative coronal alignment agreement

	Entire cohort (*n* = 258)			No recut (*n* = 140 (54%))			Recut (*n* = 118 (46%))			
	Mean ± SD ICC *n* (%)	(Range) (95% CI)	*p* values (planned vs. postoperative)	Mean ± SD ICC *n* (%)	(Range) (95% CI)	*p* values (planned vs. postoperative)	Mean ± SD ICC *n* (%)	(Range) (95% CI)	*p* values (planned vs. postoperative)	*p* values (Recut vs. No recut)
FMA										
Planned (deg)	91.7 ± 1.8	(88–96)		91.5 ± 1.9	(88–96)		91.9 ± 1.8	(88–96)		
Postoperative (deg)	91.2 ± 2.4	(84–98)	0.016	91.0 ± 2.6	(84–98)	n.s	91.4 ± 2.3	(86–98)	n.s	
Delta (deg)	− 0.5 ± 1.6	(− 9–6)		− 0.5 ± 1.7	(− 9–3)		− 0.5 ± 1.5	(− 4–6)		
Agreement	0.701	(0.617–0.767)		0.706	(0.602–0.784)		0.691	(0.572–0.780)		
Outside range [85° to 95°]	9 (3%)			4 (3%)			5 (4%)			n.s
TMA										
Planned (deg)	88.0 ± 1.3	(85–92)		88.1 ± 1.3	(85–92)		87.9 ± 1.2	(85–90)		
Postoperative (deg)	87.5 ± 2.2	(81–94)	0.002	87.8 ± 2.2	(81–94)	n.s	87.1 ± 2.1	(81–93)	< 0.001	
Delta (deg)	− 0.5 ± 1.8	(− 6–5)		− 0.2 ± 1.8	(− 6–5)		− 0.8 ± 1.8	(− 5–5)		
Agreement	0.462	(0.351–0.559)		0.479	(0.342–0.597)		0.434	(0.236–0.588)		
Outside range [85° to 95°]	21 (8%)			7 (5%)			14 (12%)			n.s
HKA angle										
Planned (deg)	179.7 ± 1.5	(176–184)		179.6 ± 1.6	(176–184)		179.7 ± 1.5	(176–183)		
Postoperative (deg)	178.6 ± 2.6	(168–185)	< 0.001	178.6 ± 2.6	(168–185	< 0.001	178.5 ± 2.6	170–185)	< 0.001	
Delta (deg)	− 1.1 ± 2.1	(− 8–5)		− 1.0 ± 1.9	(− 8–5)		− 1.3 ± 2.2	(− 8–4)		
Agreement	0.472	(0.264–0.619)		0.563	(0.347–0.704		0.368	(0.139–0.546)		
Outside range [175° to 183°]	18 (7%)			11 (8%)			7 (6%)			n.s

*FMA* femoral mechanical angle; *TMA* tibial mechanical angle; *HKA* hip knee ankle; *deg* degree; *SD* standard deviation; *ICC* intra-class correlation coefficient; *CI* confidence interval

The tibial resection level was adjusted intraoperatively by a ‘recut’ in 118 knees (46%) to achieve appropriate soft tissue balance (Table 2). The proportion of TKAs that were in the ‘target zone’ for cases that required a ‘recut’ and those that did not was, respectively, 97 (82%) and 120 (86%). Finally, the agreement between the planned and achieved targets in knees that required recuts, indicated by ICC, remained good for FMA, fair for TMA, but changed to poor for HKA angle.

## Discussion

The most important finding of the present study was that, using this strategy for coronal alignment, 84% of custom TKAs were within the ‘target zone’ in terms of FMA, TMA and HKA angles. Beyond the reliability of preoperative planning tools as well as intraoperative patient-specific instruments and techniques, the present study encompasses the main pillars for success of TKA, including personalised limb alignment, and implant geometry to reproduce bony contours and radii of curvature. The present findings support the concepts of emerging personalized medicine technologies [13, 42], and their clinical relevance emphasises the importance of detailed and accurate strategies for preoperative planning, which are key to achieving satisfactory ‘personalised alignment’ that can further be improved by customisation of implant components.

Several other findings emerge from this study. First, it outlines the limits of radiographic analysis for the understanding of patient phenotypes. The conventional 2D planning can only analyse the global limb deformity but can hardly differentiate its arthritic part (bone wear and laxity) from its constitutional part, the latter remaining unknown [6, 22, 28]. Additionally, any rotation of the leg can alter the long-leg radiograph accuracy [28]. The CT-scan allows to analyse the femur and tibia separately in their true frontal planes, and to deduce the native pre-arthritic architecture of the limb despite the bone wear. Second, this study describes a new (re)alignment strategy, based on an accurate planning, using the new possibilities offered by customization. The matrix approach relies on a rationalized ‘individual alignment’ rather than a ‘systematic alignment’ strategy and on the definition of a ‘target zone’ rather than a ‘target value’. The thresholds defining the ‘target zone’ intend to remain within safe limits in terms of tribology and fixation for the bone cuts inclination [12], the coronal asymmetry of the implants [3, 23], the joint line obliquity [32] and the realignment in valgus knees. These thresholds may be modified depending on findings in future studies. Third, it demonstrates the technical feasibility of a new process, providing a pre-manufacturing roadmap for engineers, a traceable planning for surgeons and finally individualized implants adapted to the bony anatomy and alignment. Previous series of custom TKA were based on a ‘systematic alignment’ concept intending to realign all patients to 180°, and this is the first process that aims to restore both the native shape of the bones and the constitutional alignment (CA) [3, 23].

Historically, MA has been considered as the gold standard, with several studies demonstrating its superiority for TKA survival [12, 36]. Surgeons learned how to tackle ligament imbalance due to orthogonal cuts with technical tricks such as ligament releases or external rotation of the femoral component [5], all of which are ‘palliative solutions’, compensating for the modification of the CA. Recently, several factors challenged the dogma of MA. Biomechanical studies have reported a discrepancy between the radiographic static axis of the lower limb and the functional dynamic axis during gait [40]. Large population studies demonstrated that native limb alignment is highly variable [4, 14, 30]. The rate of dissatisfaction due to residual pain or unmet expectations after TKA is reported within the range of 5–25%, [7, 8] and could be attributed to the lack of restoration of the native anatomy [4, 16, 30]. Long term studies reported that patients outside the 180° ± 3° range had similar survivorship as neutrally aligned patients [1, 11, 34], and that a residual varus may improve function [11, 27], whereas a change from varus to valgus (or vice versa) could reduce patient satisfaction [27].

**Fig. 7:**
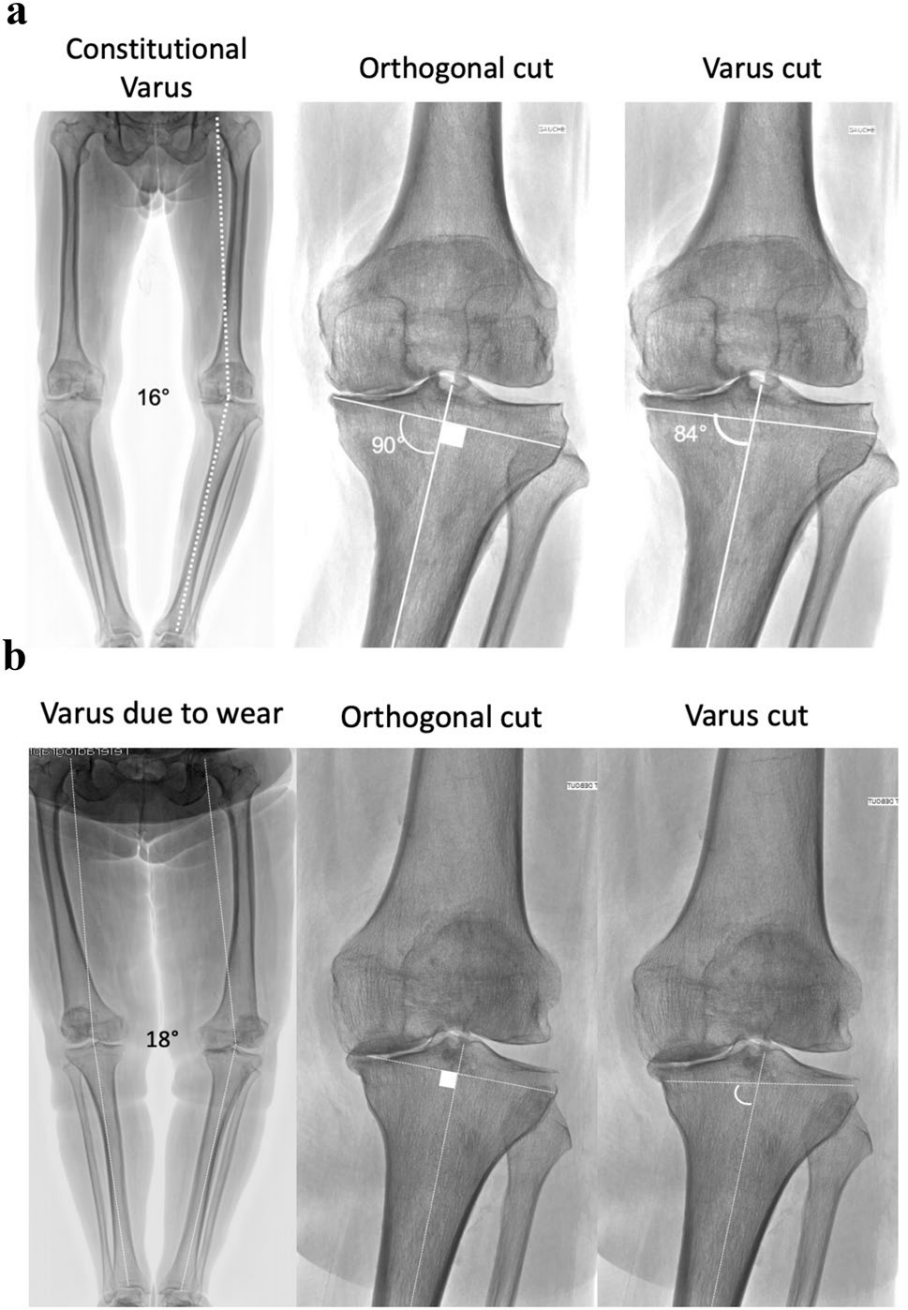
Examples where varus cuts have different consequences. In patient A, bone wear is minimal and most of the global varus deformity (16°) is due to its native architecture (constitutional varus). An orthogonal cut induce an asymmetric resection, in a weak zone of the lateral tibial plateau. With a varus cut there is less asymmetry and the bone cut remain in the subchondral area. In patient B, bone wear is severe and most of the global varus deformity (18°) is due to the bone loss (no constitutional varus). An orthogonal cut corrects the bone loss and do not induce asymmetric resection. A varus cut is very oblique with respect to the tibial shape and is situated below the subchondral area on the medial tibial plateau

Progressively, the need of a kind of personalization appeared [4, 29] and several surgeons unconsciously modified their practice from MA towards partial preservation of the preoperative deformity [26]. The epitome of this evolution is kinematic alignment (KA), where the native alignment is precisely reproduced, with a strict matching between resected bones and implant thicknesses [17]. Several authors compared MA and KA [9, 11, 44], but it is still unclear whether the differences are clinically relevant [25] and the place of KA remain controversial with three main limitations [44]. First, alignment and implant design are interrelated, and the variations of implant positioning in KA may induce bone-implant mismatch [37], trochlear malalignment [38] or femoral malrotation [33]. Second, the original KA technique is based purely on intraoperative measurements rather than on precise preoperative planning, and may be subject to surgeon experience [17]. Robotic assistance may in the future fill the gap between preoperative planning and surgical execution [9, 10]. Third, the limits of acceptable residual coronal deformity in TKA are still not clearly understood. Parratte et al. [34] and Abdel et al. [1] observed that outliers and neutrally aligned patients had similar long term survivorship, and Howell et al. [19] reported 98.4% survivorship (aseptic failures) at 10 year follow-up with KA. However, many authors report higher rates of failure in patients with residual varus [12, 27, 36]. It is worth noting that while some authors investigated the influence of residual postoperative deformities with respect to the preoperative radiographic deformity [26, 35], few investigated the role of constitutional versus arthritic deformity [43] (Fig. 7). It is also unclear whether a residual deviation has the same consequences if observed at the femur or at the tibia [27]. Based on all these unanswered questions, Almaawi et al. [2] described restricted Kinematic Alignment (rKA) which maintains a 180° ± 3° range for HKA with a 90° ± 5° range for FMA and TMA. The concept of custom TKA has also been explored with iTotal^®^ TKA (ConforMIS, Billerica, MA, USA), but using a concept of Systematic Alignment, the target being a 180° global alignment [3, 23].

The results of the present study need to be interpreted in light of the following limitations. First, the discrepancy between planned and postoperative alignment is partly attributable to the learning curve as this is a consecutive continuous series including all patients, from three senior surgeons who were trained with traditional instrumentation. Second, a tibial bone recut was performed in 46% of the procedures and a lack of accuracy of the re-cutting guides can explain discrepancies between planned and postoperative angles, particularly in patients with weak bones. Third, in this retrospective case series, there was a selection bias, since the indications for custom TKA were limited to patients without severe deformities with good preoperative ROM. Fourth, the absolute number of patients that were excluded based on the exclusion criteria, and the absolute number of patients that were excluded due to the waiting period are not reported. Finally, long term survivorship, clinical and functional outcomes are still to be confirmed; therefore, this study cannot conclude on the superiority or inferiority of custom TKA. The results do indicate the feasibility of using a more personalized strategy for coronal alignment in TKA.

## Conclusion

Using the present strategy for coronal alignment, 84% of custom TKAs were within the ‘target zone’ for FMA, TMA and HKA angles. These encouraging findings support the concepts of emerging personalized medicine technologies, and their clinical relevance emphasises the importance of detailed and accurate strategies for preoperative planning, which are key to achieving satisfactory ‘personalised alignment’ that can further be improved by customisation of implant components.

## Electronic supplementary material

Below is the link to the electronic supplementary material.

Supplementary file1 (PDF 41 kb)
