# Supplementary material for: Custom total knee arthroplasty facilitates restoration of constitutional coronal alignment
Source: Knee Surg Sports Traumatol Arthrosc. 2020 Jul 17;30(2):464–75. doi: 10.1007/s00167-020-06153-8 (PMC8866384; doi:10.1007/s00167-020-06153-8)
Supplement: Supplementary file 1 — Supplementary file1 (PDF 41 kb) [file 167_2020_6153_MOESM1_ESM.pdf]

Table: Pre-and post-operative coronal alignment according to preoperative deformity (CT) classification

		FMA (deg)						TMA (deg)						HKA angle (deg)					
		Preoperative radiograph		Preoperative CT		Postoperative radiograph		Preoperative radiograph		Preoperative CT		Postoperative radiograph		Preoperative radiograph		Preoperative CT		Postoperative radiograph	
	n	mean ±SD	(min – max)	mean ±SD	(min – max)	mean ±SD	(min – max)	mean ±SD	(min – max)	mean ±SD	(min – max)	mean ±SD	(min – max)	mean ±SD	(min – max)	mean ±SD	(min – max)	mean ±SD	(min – max)
Varus Femur																			
Varus Tibia	6	88.2 ±1.3	(86 – 89)	89.2 ±0.8	(88 – 90)	88.7 ±1.0	(87 – 90)	83.2 ±1.3	(81 – 85)	83.3 ±1.2	(81 – 84)	87.2 ±3.5	(81 – 91)	166.7 ±3.3	(162 – 172)	170.2 ±2.8	(165 – 172)	175.8 ±4.3	(168 – 180)
Neutral Tibia	30	89.5 ±1.8	(85 – 93)	89.0 ±0.9	(87 – 90)	88.9 ±2.3	(84 – 95)	86.3 ±2.5	(81 – 91)	87.2 ±1.5	(85 – 89)	88.4 ±1.8	(86 – 93)	171.6 ±4.5	(162 – 183)	173.0 ±2.3	(170 – 179)	177.3 ±2.6	(173 – 183)
Valgus Tibia	5	88.2 ±2.5	(84 – 90)	89.2 ±0.8	(88 – 90)	88.6 ±1.5	(87 – 91)	90.2 ±2.5	(87 – 94)	90.8 ±1.8	(90 – 94)	89.4 ±2.2	(87 – 93)	173.6 ±3.6	(169 – 178)	175.0 ±2.2	(172 – 178)	177.8 ±2.3	(175 – 180)
Neutral Femur																			
Varus Tibia	34	91.6 ±1.8	(88 – 95)	92.4 ±1.4	(91 – 95)	90.3 ±2.2	(85 – 94)	82.6 ±1.6	(79 – 85)	83.1 ±1.0	(80 – 84)	86.3 ±2.1	(81 – 89)	171.5 ±4.2	(166 – 184)	173.3 ±2.3	(169 – 180)	176.9 ±2.5	(170 – 181)
Neutral Tibia	120	92.3 ±1.9	(88 – 97)	92.7 ±1.4	(91 – 95)	91.3 ±1.8	(87 – 96)	86.3 ±2.0	(82 – 91)	86.9 ±1.4	(85 – 89)	87.2 ±1.9	(83 – 92)	174.9 ±5.0	(164 – 195)	176.3 ±3.8	(168 – 188)	178.3 ±1.9	(173 – 183)
Valgus Tibia	31	92.2 ±1.9	(89 – 97)	92.9 ±1.5	(91 – 95)	91.7 ±2.1	(88 – 96)	90.5 ±1.8	(87 – 95)	91.1 ±1.3	(90 – 94)	89.0 ±2.3	(84 – 94)	180.4 ±4.6	(172 – 190)	181.1 ±3.4	(174 – 188)	180.3 ±2.1	(177 – 185)
Valgus Femur																			
Varus Tibia	3	97.0 ±1.0	(96 – 98)	96.7 ±1.2	(96 – 98)	93.7 ±0.6	(93 – 94)	82.3 ±1.5	(81 – 84)	83.3 ±1.2	(82 – 84)	86.7 ±2.5	(84 – 89)	176.3 ±5.5	(171 – 182)	177.7 ±3.8	(175 – 182)	179.7 ±1.5	(178 – 181)
Neutral Tibia	23	96.1 ±1.5	(92 – 99)	97.1 ±1.2	(96 – 99)	94.3 ±1.9	(90 – 98)	87.6 ±1.8	(85 – 92)	87.3 ±1.4	(85 – 89)	86.9 ±2.1	(84 – 93)	183.1 ±4.7	(174 – 189)	183.8 ±3.6	(177 – 191)	181.1 ±2.1	(176 – 185)
Valgus Tibia	6	96.5 ±1.8	(94 – 99)	97.5 ±1.0	(96 – 99)	94.3 ±1.4	(92 – 96)	90.7 ±1.8	(88 – 93)	91.5 ±1.0	(90 – 93)	89.0 ±1.5	(87 – 91)	188.0 ±3.6	(183 – 192)	188.0 ±3.2	(184 – 192)	183.0 ±0.9	(182 – 184)

Abbreviations: FMA, femoral mechanical angle; TMA, tibial mechanical angle; HKA, hip knee ankle; deg, degree; SD, standard deviation
